# Three-dimensional *in vivo* evaluation of the cornea in patients with unilateral posterior interstitial keratitis

**DOI:** 10.3389/fmed.2023.1180208

**Published:** 2023-08-21

**Authors:** Shao-Feng Gu, Shuang Gao, Hai-Kun Wang, Lin-Hui He, Rong-Mei Peng, Ge-Ge Xiao, Jing Hong

**Affiliations:** ^1^Department of Ophthalmology, Peking University Third Hospital, Beijing, China; ^2^Beijing Key Laboratory of Restoration of Damaged Ocular Nerves, Peking University Third Hospital, Beijing, China

**Keywords:** posterior interstitial keratitis, stromal keratitis, *in vivo* confocal microscopy, anterior segment optical coherence tomography, three-dimensional evaluation

## Abstract

**Purpose:**

The purpose of this study was to investigate the *in vivo* morphologic features of the cornea in patients with unilateral posterior interstitial keratitis.

**Methods:**

Seven eyes of 7 patients with unilateral posterior interstitial keratitis were examined by slit-lamp biomicroscopy, anterior segment optical coherence tomography (AS-OCT), and *in vivo* confocal microscopy (IVCM). The imaging features of the cornea were evaluated and analyzed.

**Results:**

By slit-lamp examination, the posterior corneal stromal opacities were observed in all 7 eyes, and deep neovascularization in 4 eyes. The posterior stromal opacities showed higher reflectivity with an intact overlying epithelium by AS-OCT and did not invade the Bowman’s layer in all cases. IVCM revealed highly reflective dispersed microdots, needle-shaped bodies, and increased reflectivity of keratocytes in the lesion site in all patients. Active Langerhans cells and an attenuated subbasal nerve plexus were observed in 5 eyes. After treatment, the active Langerhans cells disappeared; however, highly reflective microdots and needle-shaped bodies remained.

**Conclusion:**

The three-dimensional evaluation of slit-lamp biomicroscopy, AS-OCT, and IVCM may help in the early diagnosis of patients with posterior interstitial keratitis.

## Introduction

Interstitial keratitis (IK), also known as immune stromal keratitis (ISK), is a nonulcerative, non-suppurating stromal immune reaction without primary involvement of the corneal epithelium or endothelium (1). This is a clinical diagnosis, and slit-lamp examination may reveal infiltration in the anterior, mid, or posterior stroma. A variety of causes of interstitial keratitis have been elucidated, including syphilis, viral infection, and Cogan syndrome. Currently, herpes virus infection is the primary cause of interstitial keratitis in North America (2–4).

Posterior interstitial keratitis is a form of interstitial keratitis in which infiltration localizes to the posterior stroma, with or without corneal neovascularization, thinning of the corneal stroma, and other signs (5). Previous studies have shown that stromal infiltration of interstitial keratitis is a form of chronic recurrent inflammation that can persist for years, and stromal opacities most likely represent antigen–antibody-complement immune complexes (2, 5). Mild interstitial keratitis may have nebulous scars and faint ghost vessels; severe cases may leave prominent vessels, a dense scar, and corneal thinning, which may need keratoplasty to improve vision. Patients with posterior interstitial keratitis are usually asymptomatic or have little discomfort, and it is easy to misinterpret active inflammation for quiescent scarring of the cornea (6). Therefore, early diagnosis is likely to improve the outcome of posterior interstitial keratitis.

Among the most ubiquitous clinical instruments, slit-lamp biomicroscopy is the gold standard in clinical practice for evaluating corneal inflammation with a wide range of magnifications and forms of illumination. However, involuntary eye movements, less resolution and/or depth of field, and a small range of magnification and illumination may limit the quality of the image. Anterior segment optical coherence tomography (AS-OCT) provides noncontact *in vivo* corneal cross-sectional, high-resolution views that elucidate the structural details of various corneal pathologies, and it can directly visualize, objectively measure, and assess *in vivo* parameters of corneal lesions (7). Limitations of this diagnostic imaging technology are the impossibility of measuring the density and imaging to the level of individual cells (8, 9). *In vivo* confocal microscopy (IVCM) allows real-time, noninvasive, high magnification, *in vivo* observation of normal and pathologic corneal microstructures at the cellular level, and it has become an increasingly popular choice for the *in vivo* assessment of corneal disorders (10, 11). The disadvantage of this technology includes the limited field of view and 2D grayscale images, which cannot substitute for histopathologic diagnosis.

AS-OCT and IVCM have been used to study numerous corneal diseases (6, 12). However, to our knowledge, there are few reports in the ophthalmic literature describing the characteristics of the cornea by AS-OCT and IVCM in patients with posterior interstitial keratitis. As mentioned above, slit-lamp biomicroscopy could reveal the location and appearance of the lesions, and provide general dimensions. AS-OCT showed the range and depth of the lesions, which revealed cross-section structural details of the lesions. This is the cross-section dimension. IVCM could elucidate the axon plane structural details of the lesions at the cellular level, and this is the axon plane dimension. The combination of slit lamp biomicroscopy, AS-OCT, and IVCM could provide the three-dimensional evaluation of posterior interstitial keratitis. The aim of the present study was to investigate the three-dimensional characteristics of the cornea with posterior interstitial keratitis by using slit-lamp biomicroscopy, AS-OCT, and IVCM, which may help with early diagnosis of posterior interstitial keratitis.

## Patients and methods

### Participants

This was an observational, retrospective, descriptive case series study. Seven eyes of 7 patients with unilateral posterior interstitial keratitis were diagnosed and treated between January 2015 and December 2021 at the Department of Ophthalmology, Peking University Third Hospital. The clinical diagnosis was performed based on a clinical presentation in which the inflammation was localized to the posterior stroma, and there were no overlying epithelial defects. This research followed the tenets of the Declaration of Helsinki and was approved by the Institutional Ethics Committee of the Ophthalmic Research Center, affiliated with Peking University Third Hospital, Beijing, China (number M2021283). An informed consent form was obtained from all participants.

Anterior chamber paracentesis was performed on all patients using an aseptic technique (after informed consent was obtained) during the first visit to our clinic. Herpes simplex virus (HSV), herpes zoster virus (HZV), varicella-zoster virus (VZV), cytomegalovirus (CMV), and Epstein–Barr virus (EBV) infection were investigated by real-time polymerase chain reaction (RT–PCR) analysis of aqueous humor samples from the affected eye. In addition, the corneal tissue sample of case 6 was analyzed by using metagenomic sequencing during deep lamellar keratoplasty. Demographic characteristics, ocular and medical history, laboratory investigations, and treatment regimens were reviewed. Ophthalmic examinations included visual acuity assessment, tonometry, and dilated fundus examination.

### Slit-lamp photography and AS-OCT examination

All patients were examined and photographed using a slit-lamp biomicroscope (Haag-Streit BM900; Haag-Streit USA, Inc., Mason, Ohio) by experienced technicians at each visit. The lesion sites were analyzed by AS-OCT (Visante, Carl Zeiss Meditec, Inc., Dublin, CA). In addition, the central corneal thickness (CCT) and the corneal thickness at the site of the lesion (CTL) were also measured by AS-OCT. The same operator adjusted the software system to position the vertex at the center of the AS-OCT image. The thickness was measured using the software on the Visante AS-OCT. The CCT was measured with the caliper position at zero and recorded as the distance from the surface epithelium to the endothelium. CTL evaluation was performed manually at the site of the lesions. Cursors were placed perpendicular to the anterior corneal surface at the point of measurement.

### *In vivo* confocal microscopy

Confocal images were captured using an HRT 3 RCM confocal microscope (HRT3/RCM, Heidelberg Engineering, Heidelberg, Germany). Under manual control of the x–y position of the image and the section depth, serial sections of the lesion site were examined and recorded. A clear image of each layer (the basal epithelium, Bowman’s membrane, anterior stroma, posterior stroma, and endothelium) was selected for analysis. To calculate cell density, at least 50 cells in a counting frame size were included for each analysis. The calculation was performed after the cells were manually marked. The system’s proprietary software calculated the number of cells per square millimeter. The results are expressed as cells/mm^2^.

### Statistical analysis

Statistical analyses were performed with SPSS Statistics for Windows, version 21.0 (IBM Corp., Armonk, NY, United States). Data are expressed as the mean ± standard deviation (SD) and range.

## Results

### Patient demographics

The mean age of the patients was 37 ± 9.5 years (range 23–48 years). Six (86%) were female, and 1 (14%) was male. There were more right eyes (71.4%) than left eyes (28.6%). All patients had unilateral involvement with no significant past medical complications. Patients 1, 2, 4, and 7 had no notable past ocular history or antivirals history. Patients 3, 5, and 6 had a history of oral or topical antiviral agents. Patient 3 was diagnosed with dendritic epithelial keratitis 6 years ago and managed with topical antivirals by an outside provider (the details were unclear). Patient 5 was diagnosed with herpetic keratitis 25 years ago and treated with topical antivirals subconjunctivally by an outside ophthalmologist (details were unclear). Patient 6 was diagnosed with herpetic keratitis and treated with oral antivirals by an outside ophthalmologist 2 years ago (the details were unclear).

A review of systems, medical history, and social history excluded *Borrelia burgdorferi* infections (none had visited endemic areas) and Cogan syndrome (no tinnitus or hearing loss) in all patients. *Treponema pallidum* serology revealed that the patients were negative for this infection. None of the analyzed aqueous humor samples demonstrated the presence of HSV, HZV, VZV, CMV, or EBV DNA. However, HSV was identified in the corneal sample of patient 6 by metagenomic sequencing. The demographic data and clinical features of the individuals are summarized in Table 1.

**Table 1:** Clinical data and slit-lamp biomicroscopic findings of patients with unilateral posterior interstitial keratitis.

Case no.	Age (years)	Sex	Eye	Ocular history	BCVA (before/after treatment)	IOP (mmHg)	Slit-lamp findings	Corneal NV	KPs and AC cells	RT–PCR results	Duration (months)
1	39	F	Right	unremarkable	0.699/0.201	8–14	Ground-glass haze, central	(−)	(+)	AH (−)	23
2	25	F	Right	unremarkable	0.201/0.097	16–20	Ground-glass haze, paracentral	(−)	(−)	AH (−)	7
3	48	F	Left	dendritic epithelial keratitis	0.097/0	17–19	“Steel wool-like” haze, peripheral, multiple	(−)	(−)	AH (−)	5
4	23	F	Left	unremarkable	0.699/0.398	11–23	“Multiple snowflake-like” haze, diffuse	(+)	(+)	AH (−)	24
5	41	F	Right	herpetic keratitis	0/0	18–20	Gray punctate haze, peripheral, multiple	(+)	(−)	AH (−)	36
6	45	F	Right	herpetic keratitis	0.886/0.495	17–20	Gray punctate haze, paracentral	(+)	(+)	AH (−)	15
7	37	M	Right	unremarkable	0.201/0	11–14	Ground-glass haze, paracentral	(+)	(+)	AH (−)	18

BCVA, best corrected visual acuity (logarithm of the minimum angle of resolution); IOP, intraocular pressure; NV, neovascularization; KPs, keratic precipitates; AC, anterior chamber; RT–PCR, real-time polymerase chain reaction; AH, aqueous humor.

### Slit-lamp examination findings

**Figure 1:**
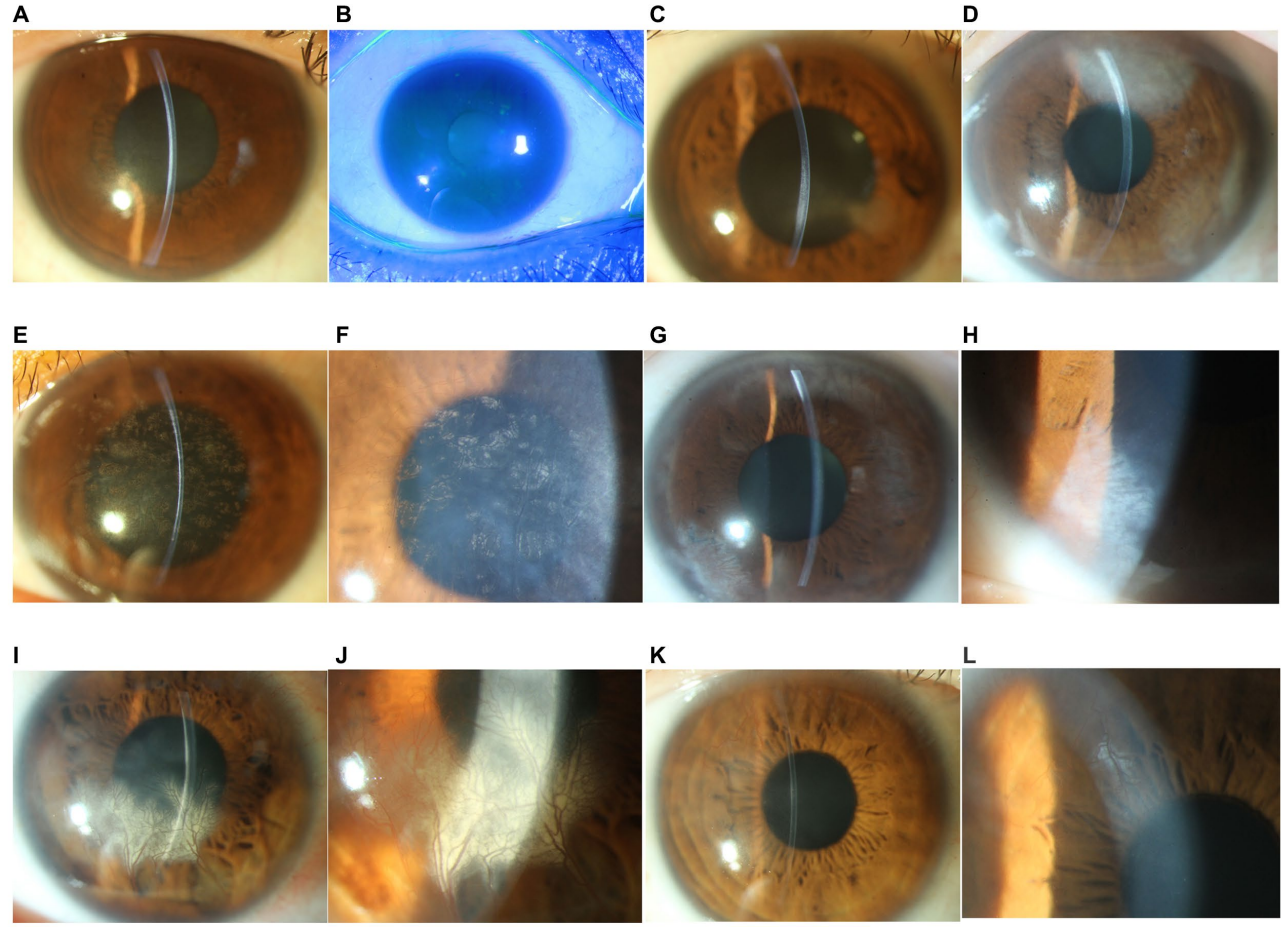
Slit-lamp photograph of patients with unilateral posterior interstitial keratitis. **(A)** The right eye of patient 1 demonstrated stromal opacities in the posterior stroma (16×). **(B)** Fluorescein staining of the same eye was negative (10×). **(C)** The right eye of patient 2 demonstrated stromal opacities in the posterior stroma and an abnormal posterior curvature (16×). **(D)** The left eye of patient 3 demonstrated a peripheral posterior stromal “steel wool-like” haze (16×). **(E)** The left eye of patient 4 showed diffuse posterior stromal “snowflake-like” haze and neovascularization (16×). **(F)** Local magnification of the same eye (25×). **(G)** The right eye of patient 5 demonstrated posterior multiple stromal haze and neovascularization (16×). **(H)** Local magnification of the same eye (25×). **(I)** The right eye of patient 6 demonstrated inferior posterior stromal haze and neovascularization (16×). **(J)** Local magnification of the same eye (25×). **(K)** The right eye of patient 7 demonstrated paracentral posterior stromal haze and superior neovascularization. **(L)** Local magnification of the same eye (25×).

All patients were either asymptomatic or had only mild blurring or photophobia at presentation. Slit-lamp examination revealed posterior corneal stromal opacities in all cases, deep neovascularization in 4 eyes (patients 4, 5, 6, and 7; 57.1%), and mild dusty keratic precipitates (KPs) and mild anterior chamber cells (less than 1+) in 4 eyes (patients 1, 4, 6, and 7; 57.1%). The otherwise was normal, including white and quiet conjunctiva, negative fluorescence, and a deep anterior chamber. The slit-lamp photographs of all patients are shown in Figure 1.

A presumptive diagnosis of herpetic keratitis was made, and all patients were treated with oral antivirals and topical corticosteroids. After treatment, all patients had an improved clinical appearance, and best corrected visual acuity (BCVA) improved in 6 eyes (patients 1, 2, 3, 4, 6, and 7; 86%). KPs and anterior chamber cells disappeared in all 4 eyes. However, deep neovascularization remained.

### AS-OCT findings

**Figure 2:**
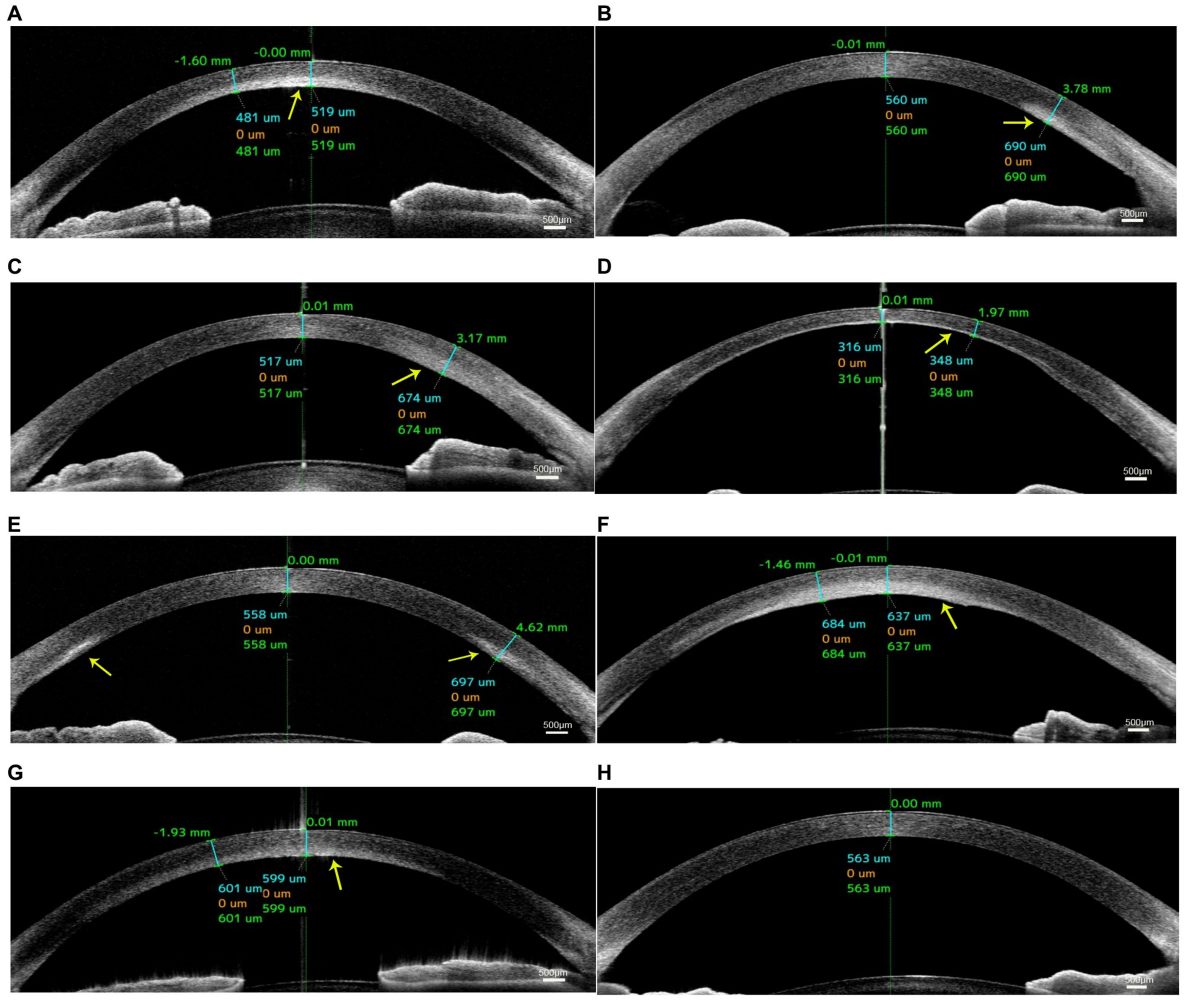
Anterior segment optical coherence tomography (AS-OCT) images of patients with unilateral posterior interstitial keratitis. OCT images of patient 1 **(A)**, patient 2 **(B)**, patient 3 **(C)**, patient 4 **(D)**, patient 5 **(E)**, patient 6 **(F)**, and patient 7 **(G)** showed hyperreflectivity in the lesion area of the posterior stroma (arrow). **(H)** An image of the normal tear film and cornea.

AS-OCT images demonstrated higher reflectivity of the posterior stroma in the lesion site than in the unaffected area in all cases. The higher reflectivity was limited in the posterior stroma and did not invade the Bowman’s layer with an intact overlying epithelium in all cases (Figure 2). The corneal shape of 5 eyes (patients 2, 3, 5, 6, and 7; 71.4%) was thickened within the affected zones. The mean CCT was 567.4 ± 45.7 μm, and the mean CTL was 674.3 ± 158.7 μm. After treatment, the mean CCT decreased to 504.0 ± 87.9 μm, and the mean CTL decreased to 542.6 ± 114.3 μm. The details are shown in Table 2.

**Table 2:** Mean pachymetry measurements by AS-OCT of in patients with unilateral posterior interstitial keratitis.

	Before treatment		After treatment	
Case no.	CCT (μm)	CTL (μm)	CCT (μm)	CTL (μm)
1	548	568	519	481
2	560	690	526	526
3	580	990	517	674
4	490	490	316	348
5	558	697	555	668
6	637	684	591	591
7	599	601	504	510
Mean ± SD	567.4 ± 45.7	674.3 ± 158.7	504.0 ± 87.9	542.6 ± 114.3

CCT, central corneal thickness; CTL, corneal thickness at the lesion site; AS-OCT, anterior segment optical coherence tomography.

### IVCM findings

**Figure 3:**
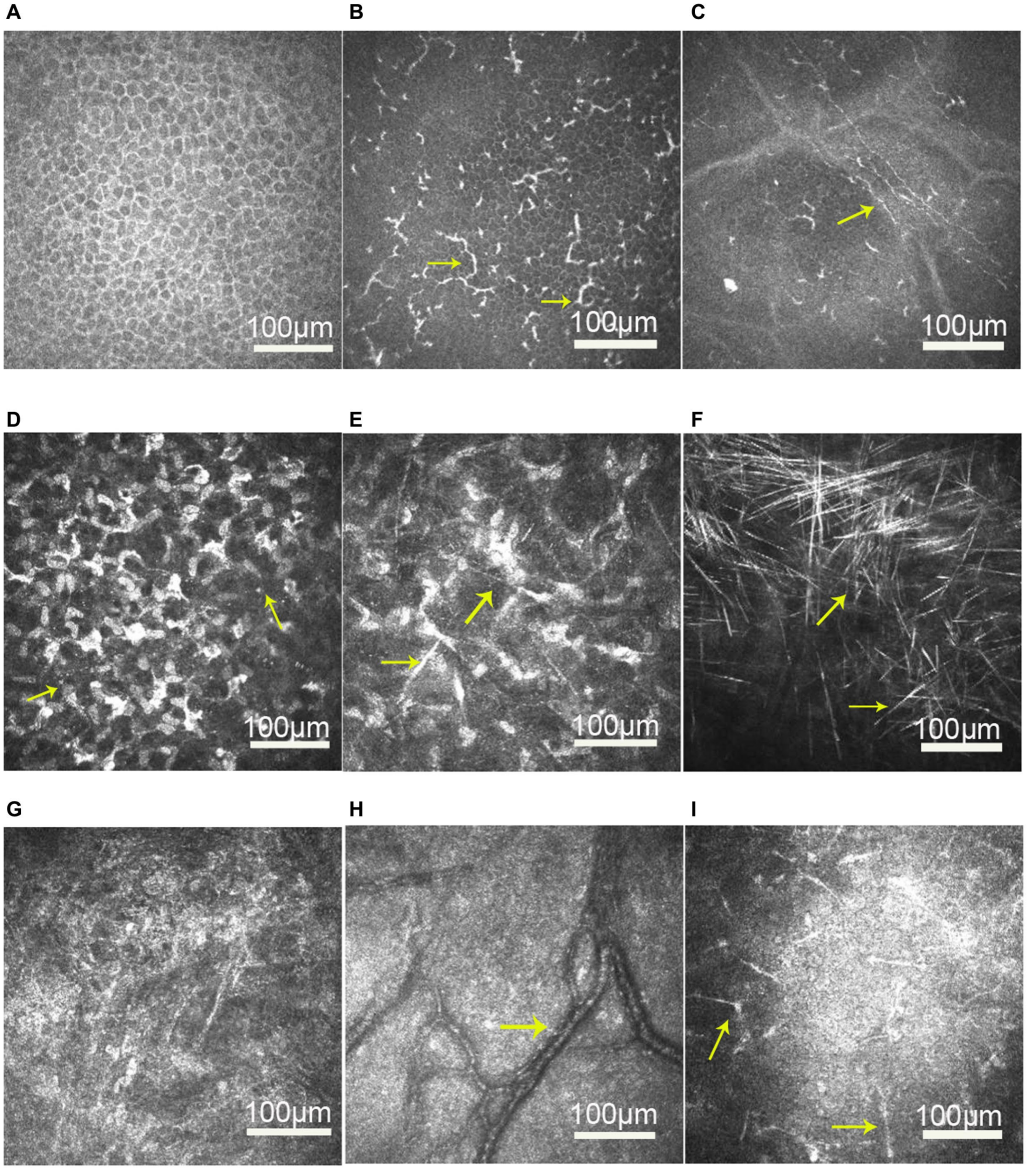
*In vivo* confocal microscopy images of the cornea in patients with unilateral posterior interstitial keratitis. **(A)** Slightly increased cell body size and intercellular space in the epithelial layers of patient 6. **(B)** Dendritic cells increased in the epithelial basal cell layer of patient 6 (arrow). **(C)** Nerve density was reduced in patient 1. **(D)** Highly reflective dispersed microdeposits were observed in the corneal stroma of patient 6 (arrow). **(E)** Highly reflective needle-shaped bodies and increased reflectivity of keratocytes were observed in the corneal stroma of patient 1 (arrow). **(F)** Needle-shaped bodies were more obvious in patient 3 (arrow). **(G)** Disorganized collagen lamellae in patient 1. **(H)** Vessels were observed in the posterior stroma in patient 6 (arrow). **(I)** Diffusely distributed dendriform keratic precipitates (KPs) were observed on the endothelium in patient 1 (arrow).

IVCM revealed normal epithelial layers in 5 patients (patients 1, 2, 3, 4, and 5; 71.4%) and slightly increased cell body size and intercellular space in 2 patients (patients 6 and 7; 28.6%; Figure 3A). The infiltration of the basal epithelial layers with inflammatory cells was evident in 5 eyes (patients 1, 2, 3, 6, and 7; 71.4%; Figure 3B) in the form of dendritic cells. The subbasal nerve plexus appeared attenuated in 5 eyes (patients 1, 2, 3, 6, and 7; 71.4%; Figure 3C) and remained normal in 2 eyes (patients 4 and 5; 28.6%). Highly reflective dispersed microdots, needle-shaped bodies, and increased reflectivity of keratocytes were observed in the corneal stroma of all patients (6/6; 100%; Figures 3D,E). Needle-shaped bodies were more obvious in patient 3 (14%; Figure 3F). In addition, the posterior stromal architecture was distorted with disorganized collagen lamellae in 5 eyes (patients 1, 3, 4, 6, and 7; 71.4%; Figure 3G). Vessels were noticed in the posterior stroma in 4 eyes (patients 4, 5, 6, and 7; 57.1%; Figure 3H), and dendriform KPs were observed on the endothelium in 4 eyes (patients 1, 4, 6, and 7; 57.1%; Figure 3I).

After treatment, the epithelial cells became normal, and active dendritic cells and KPs disappeared in all cases. However, highly reflective microdots, needle-shaped bodies, and distorted posterior stromal collagen lamellae remained. The mean endothelial cell density of all cases decreased from 2494.4 ± 173.4 cells/mm^2^ to 2279.0 ± 274.5 cells/mm^2^.

## Discussion

Posterior interstitial keratitis, a clinical diagnosis characterized as an inflammatory lesion localized at the posterior corneal stroma by slit-lamp examination, has rarely been described in the ophthalmic literature. This clinical presentation has been seen with both infectious and noninfectious diseases, and an appropriate differential diagnosis is important. In 2018, Farooq described a case series with the clinical pattern of posterior interstitial keratitis and suggested that posterior interstitial keratitis, particularly with the unilateral presentation, “steel wool-like” stromal haze, and/or previous dendritic epithelial keratitis, should be presumed to be caused by HSV (13). In this study, we investigated 7 eyes with posterior interstitial keratitis and found clinical ophthalmologic manifestations similar to those described by Farooq. Two patients had a history of herpetic keratitis, and one had a history of epithelial keratitis. However, the aqueous humor samples were negative for HSV, HZV, VZV, CMV, and EBV in all cases. Only HSV was identified in the cornea sample of patient 6 by metagenomic sequencing. We made a presumptive diagnosis of herpetic keratitis and treated the patient with oral antivirals and topical corticosteroids. All patients had an improved clinical appearance, and visual acuity improved in most cases. Previous studies have shown that recurrent HSV interstitial keratitis is related to viral activity and the host immune response. Some researchers have hypothesized that long-term viral antigen persistence occurs in the corneal stroma (14, 15). Thus, the low viral load in aqueous humor samples may be the reason for the negative RT–PCR results. In addition, we noticed females were more than male patients. Klein reported that the responses to viral infections between males and females were different (16). The heightened antiviral, inflammatory, and immune responses in females might underlie the increased development of symptoms of disease, which might be why the more female patients in the study.

AS-OCT was used to observe changes in corneal structure, and we found higher reflectivity in these lesions of the posterior corneal stroma in all patients. Higher reflectivity was limited in the posterior stroma. Five eyes showed a thicker posterior stroma than unaffected areas. After treatment, the thickness of the cornea was thinned, and the mean CCT was less than 550 μm. Previous studies have suggested that inflammatory infiltration appeared as a high reflectivity area with an increased stromal thickness (17), and corneal thinning may occur as a sequela of the inflammatory process due to fibrosis and scarring (2), potentially underlying the corneal thickness decrease observed in this study.

All patients’ corneas were subjected to IVCM to investigate the microstructural changes in posterior interstitial keratitis. We observed active Langerhans cells in the form of dendritic cells below the basal epithelium. Langerhans cells, which are antigen-presenting cells, are usually present in the peripheral cornea and can migrate to areas of inflammation. Mastropasqua et al. (18) suggested that dendritic cells were excellent indicators of inflammatory activity. In addition, we noted a decreased density of subbasal nerves in 5 eyes (71.4%). After treatment, the dendritic cells disappeared from the central cornea, but the density of the subbasal nerves remained attenuated until the last follow-up in all 5 cases. Previous studies have demonstrated an increase in corneal dendritic cells with a decreased subbasal nerve plexus in infectious keratitis and suggested that any inflammatory process could potentially lead to loss of corneal innervation and this interplay may not be disease-specific (19, 20). It has been postulated that there was a connection between the immune and nervous systems through the interaction of cytokines and interleukins produced by leukocytes to receptors expressed on nerves and cells of the neuroendocrine system (21).

In addition to the above-described morphologic changes, we noticed highly reflective dispersed microdots, needle-shaped bodies, and increased reflectivity of keratocytes in the corneal stroma of all patients. Microdots are considered a degenerative corneal disease, and their incidence increases with contact lens wear and some keratopathy (e.g., Thygeson’s superficial punctate keratopathy) (22). The precise origin of microdots remains obscure. It has been hypothesized that they may be lipofuscin-like material that accumulates as a result of oxidative stress due to chronic hypoxia of the cornea (23). Needle-shaped bodies are also known as crystals and can be caused by a variety of conditions, including lecithin cholesterol acyltransferase deficiency (24) and chronic herpetic keratitis (25). The pathogenesis of the crystals has also not been identified. However, studies postulated that they might comprise depositions of immune complexes resulting from a localized immune reaction or originating from the passage of lipids from the vasculature due to chronic low-grade inflammation (26).

Dendriform KPs were observed on the endothelium in four eyes and disappeared quickly after treatment. A mild decrease in the mean endothelial cell density was found in all cases. Inflammation of the posterior corneal stroma and concomitant endothelitis may be the reason for this decrease. Therefore, early diagnosis and treatment to resolve active corneal posterior stroma inflammation may be important to reduce the risk of damage to endothelial cells.

This study has several limitations. First, this was an observational and retrospective study with small-size sample. The aqueous humor samples were negative for HSV, HZV, VZV, CMV, and EBV in all cases by RT–PCR. In the future, a prospective study involving a larger cohort of patients with advanced molecular techniques and histological examination of the cornea may enable the identification of the etiology. Second, new innovative techniques in ophthalmology are expected to allow more precise visualization of corneal structures, which can improve the elucidation of clinical features of posterior interstitial keratitis in the future. For example, wide-field and micron-resolution visible light optical coherence tomography (vis-OCT) expands the imaging capabilities of vis-OCT (27), and inverse spectroscopic optical coherence tomography (IS-OCT) can detect nanoscale ultrastructural changes in the cornea (28).

In conclusion, our study revealed that by slit-lamp biomicroscopy and AS-OCT, the lesion of posterior interstitial keratitis may be limited to the posterior corneal stroma. However, IVCM showed that the inflammatory reaction might involve the whole cornea. It is essential to differentiate this active keratitis from other innocuous keratopathies. Changes in the appearance of the cornea could be observed by slit-lamp biomicroscopy; changes in the corneal tissue structure were visible by AS-OCT, and changes in the corneal cells could be investigated by IVCM. Therefore, the combination of slit-lamp biomicroscopy, AS-OCT, and IVCM may permit a three-dimensional evaluation of the morphological characteristics of the cornea in patients with posterior interstitial keratitis. This approach could be useful for the early diagnosis and monitoring of the therapeutic effects of drugs.

## Data availability statement

The original contributions presented in the study are included in the article/supplementary material, further inquiries can be directed to the corresponding author.

## Ethics statement

The studies involving human participants were reviewed and approved by Institutional Ethics Committee of the Ophthalmic Research Center, affiliated with Peking University Third Hospital, Beijing, China. The patients/participants provided their written informed consent to participate in this study. Written informed consent was obtained from the individual(s) for the publication of any potentially identifiable images or data included in this article.

## Author contributions

S-FG, SG, H-KW, L-HH, R-MP, G-GX, and JH contributed to the study’s conception and design. Material preparation was performed by L-HH, R-MP, and G-GX. Data collection and analysis were performed by S-FG, SG, and H-KW. The first draft of the manuscript was written by S-FG. Writing-review and funding acquisition was performed by JH and R-MP. All authors contributed to the article and approved the submitted version.

## Funding

This research project was supported by the China National Key Research and Development Program under grant no. 2020AAA0105004 and the National Natural Science Foundation of China under grant nos. 81970768 and 81800801. The funding organization had no role in the design or conduct of this research.

## Conflict of interest

The authors declare that the research was conducted in the absence of any commercial or financial relationships that could be construed as a potential conflict of interest.

## Publisher’s note

All claims expressed in this article are solely those of the authors and do not necessarily represent those of their affiliated organizations, or those of the publisher, the editors and the reviewers. Any product that may be evaluated in this article, or claim that may be made by its manufacturer, is not guaranteed or endorsed by the publisher.
